# Neural Correlates of Opposing Effects of Emotional Distraction on Working Memory and Episodic Memory: An Event-Related fMRI Investigation

**DOI:** 10.3389/fpsyg.2013.00293

**Published:** 2013-06-06

**Authors:** Florin Dolcos, Alexandru D. Iordan, James Kragel, Jared Stokes, Ryan Campbell, Gregory McCarthy, Roberto Cabeza

**Affiliations:** ^1^Psychology Department, University of Illinois at Urbana-Champaign, Urbana, IL, USA; ^2^Neuroscience Program, University of Illinois at Urbana-Champaign, Urbana, IL, USA; ^3^Beckman Institute for Advanced Science and Technology, University of Illinois at Urbana-Champaign, Urbana, IL, USA; ^4^Center for Cognitive Neuroscience, Duke University, Durham, NC, USA; ^5^Department of Psychological Sciences, Vanderbilt University, Nashville, TN, USA; ^6^Department of Psychology, University of California Davis, Davis, CA, USA; ^7^Department of Psychiatry, University of Alberta, Edmonton, AB, Canada; ^8^Brain Imaging and Analysis Center, Duke University, Durham, NC, USA; ^9^Department of Psychology, Yale University, New Haven, CT, USA

**Keywords:** emotional interference, emotional control, emotional memory, AMY, HC, vlPFC, dlPFC

## Abstract

A fundamental question in the emotional memory literature is why emotion enhances memory in some conditions but disrupts memory in other conditions. For example, separate studies have shown that emotional stimuli tend to be better remembered in long-term episodic memory (EM), whereas emotional distracters tend to impair working memory (WM) maintenance. The first goal of this study was to directly compare the neural correlates of EM enhancement (EME) and WM impairing (WMI) effects, and the second goal was to explore individual differences in these mechanisms. During event-related functional magnetic resonance imaging (fMRI), participants maintained faces in WM while being distracted by emotional or neutral pictures presented during the delay period. EM for the distracting pictures was tested after scanning and was used to identify successful encoding activity for the picture distracters. The first goal yielded two findings: (1) emotional pictures that disrupted face WM but enhanced subsequent EM were associated with increased amygdala (AMY) and hippocampal activity (ventral system) coupled with reduced dorsolateral PFC (dlPFC) activity (dorsal system); (2) trials in which emotion enhanced EM without disrupting WM were associated with increased ventrolateral PFC activity. The ventral-dorsal switch can explain EME and WMI, while the ventrolateral PFC effect suggests a coping mechanism. The second goal yielded two additional findings: (3) participants who were more susceptible to WMI showed greater amygdala increases and PFC reductions; (4) AMY activity increased and dlPFC activity decreased with measures of attentional impulsivity. Taken together, these results clarify the mechanisms linking the enhancing and impairing effects of emotion on memory, and provide insights into the role of individual differences in the impact of emotional distraction.

## Introduction

Emotion is a “double-edged sword” that can either enhance or hinder various aspects of our cognition and behavior. For instance, the emotional charge of an event can lead to better episodic memory (EM) for that event, whereas task-irrelevant emotional distraction can impair working memory (WM), if presented concurrently with goal-relevant information (reviewed in Dolcos et al., 2011; Dolcos et al., 2012; Iordan et al., 2013b; also see Cohen and Henik, 2012 in the present research topic). Although previous research has independently investigated these two opposing effects of emotion on EM and WM, very little is known about their interactions and the associated neural mechanisms. Concomitant investigation of enhancing and impairing effects of emotion and of their interaction is important because they tend to co-occur. For instance, hearing a gunshot may enhance memory for central aspects of what was happening at the time, while impairing memory for peripheral details (Christianson and Loftus, 1991; Kensinger et al., 2007; also see Chiu et al., 2013 in the present research topic). On the other hand, increased distraction from on-going goals produced by task-irrelevant emotional stimuli may also lead to better memory for the distracting information. The present study directly compared the neural mechanisms of EM enhancing (EME) and WM impairing (WMI) effects of emotion, by using a novel paradigm that measured both the initial impact of emotional distraction on WM and the long-term EM for the distracters themselves. The study also investigated the role of individual differences in these effects. Below, we briefly review the available evidence concerning the EME and WMI effects of emotion, as derived from their separate investigation, and introduce the rationale for the present approach.

### The link between opposing effects of emotional distraction on WM and EM

In both EM and WM literatures, emotion effects have been interpreted in terms of bottom-up and top-down systems. Bottom-up systems are assumed to be relatively automatic and guided by the stimuli, whereas top-down systems are assumed to be controlled and guided by task goals. In the EM literature, the bottom-up and top-down systems have been described as direct vs. indirect and a collaborative relationship has been emphasized, whereas in the WM literature, the bottom-up and top-down systems have been described as *hot* vs. *cold* and the findings have shown an opposing relationship. Importantly, these dissociations also map onto similar ventral and dorsal neural systems, but it is unclear to what extent they overlap or are dissociable.

Available evidence from us and others regarding the EME effect of emotion suggests the existence of two neural routes (reviewed in LaBar and Cabeza, 2006; Dolcos et al., 2011, 2012). Briefly, one route (direct/bottom-up), consisting of emotion-based (amygdala, AMY) and memory-based (hippocampus, HC) medial-temporal lobe (MTL) structures, is thought to operate more automatically and largely independently of resources at the time of encoding (Dolcos et al., 2004b; Shafer and Dolcos, 2012). The other route (indirect/top-down), involving prefrontal and parietal cortices (PFC and PC, respectively), is thought to depend on the contribution of other processes to the memory-enhancing effect of emotion, such as semantic memory, executive control, and attention (Dolcos et al., 2004a). Of note, the evidence supporting the dissociation between these two routes also maps onto a ventral/dorsal location of the associated neural correlates – AMY-HC vs. PFC/PC, respectively. Consistent with this dissociation, recent evidence identified AMY-HC contribution (bottom-up/ventral) to emotional EME following a shallow level of processing during encoding, and the engagement of cognitive control areas (top-down/dorsal) under a deep level of processing (Ritchey et al., 2011). Similarly, evidence from a recent study by Shafer and Dolcos (2012), investigating the link between the immediate and long-term impact of emotional distraction, identified bottom-up/ventral (AMY-HC) mechanisms contributing to EME by emotion, in conditions of limited resources available during encoding. Overall, the available evidence concerning the EME effect points to contributions of both direct/bottom-up/ventral and indirect/top-down/dorsal mechanisms.

Turning to the WMI effect of emotional distraction, a series of functional magnetic resonance imaging (fMRI) studies by Dolcos et al. and studies by others (Dolcos and McCarthy, 2006; Dolcos et al., 2006, 2008; Anticevic et al., 2010; Chuah et al., 2010; Denkova et al., 2010; Iordan et al., 2013a; reviewed in Iordan et al., 2013b) shed light on the neural mechanisms underlying the impact of transient emotional distraction on WM maintenance. Interestingly, similar to the EME effect of emotion, these studies also identified a ventral-dorsal dissociation in the neural correlates of the WMI effect of emotional distraction. Using an experimental design where task-irrelevant emotional distracters were presented during the delay interval of a WM task, these studies demonstrated that the impairing effect of emotional distraction was linked to opposing patterns of activity in brain regions associated with a ventral neural system involved in *hot* emotional processing (*HotEmo* system) and a dorsal neural system associated with *cold* executive processing (*ColdEx* system) (reviewed in Dolcos et al., 2011). Specifically, emotional distraction enhanced activity in ventral-affective regions, such as the AMY, while disrupting delay activity in dorsal-executive regions, such as the dorsolateral PFC (dlPFC) and the lateral parietal cortex (LPC). Given the role of the latter brain regions in attentional processes and active maintenance of goal-relevant information in WM (D’Esposito et al., 2000; Hopfinger et al., 2000; Levy and Goldman-Rakic, 2000; Miller and Cohen, 2001), these findings suggest that activity in the affective and executive neural systems is strongly interconnected, such that increased activity in the ventral-affective regions disrupts activity in the dorsal-executive system and results in WM impairment, possibly as a result of a re-allocation of processing resources by emotional distraction (Dolcos and McCarthy, 2006). Noteworthy, the studies investigating the WMI effect of emotion also identified the neural correlates of coping with emotional distraction (Dolcos and McCarthy, 2006; Dolcos et al., 2006, 2008; Chuah et al., 2010; Denkova et al., 2010; Iordan et al., 2013a; reviewed in Iordan et al., 2013b), and highlighted the role of both basic emotion processing regions (AMY) and regions involved in cognitive control (PFC). In this network, AMY presumably signals PFC regions about the presence of emotional, potentially distracting, stimuli, and thus the need to engage cognitive control mechanisms to cope with emotional distraction (Dolcos et al., 2006, 2008; Chuah et al., 2010; Denkova et al., 2010).

Given the lack of evidence linking these opposing effects of emotion, the first goal of the present study was to directly compare the neural mechanisms of EME and WMI effects. The evidence discussed above identified the involvement of both bottom-up/ventral and top-down/dorsal mechanisms involved in the EME and WMI effects of emotion. What remains unclear, however, is the link between these two opposing effects and the role of the associated neural correlates. Specifically, it is unclear how the initial response to emotional distraction, leading either to impairment or to coping in the presence of task-irrelevant emotional stimuli, influences the long-term memory for this potentially distracting information, and what the neural mechanisms linking the immediate and long-term effects of distracting emotions are. Of particular importance is identification of the role of both ventral and dorsal brain areas that have been commonly identified by the separate investigations of the EME and WMI effects of emotion – i.e., AMY-MTL and PFC.

### The role of individual differences in the impact of emotional distraction

The second goal of the present investigation concerns the role of individual differences in the relationship between the enhancing and impairing effects of emotion. This is justified by evidence that, in addition to general emotion processing, both EME and WMI effects of emotion, along and with the engagement of coping strategies are susceptible to individual variations (Canli et al., 2002; Hamann and Canli, 2004; Touryan et al., 2007; Dolcos et al., 2008; Hooker et al., 2008; Iordan et al., 2013a). This suggests that differences that affect both ventral/bottom-up and dorsal/top-down mechanisms, involved in emotional and cognitive/executive processing, can influence the initial impact of emotional distraction on WM (Dolcos et al., 2008; Iordan et al., 2013a) and possibly the relationships between the WMI and EME effects. Of particular relevance for the present investigation is evidence from a recent study showing that, while in most participants emotional distraction impaired WM performance, in some subjects it did not have a detrimental effect (Dolcos et al., 2008), thus pointing to individual variation in the susceptibility to emotional distraction. However, because that study did not involve assessments of participants in cognitive and emotional domains other than related to the WM task and emotional ratings, it is not clear why some participants were more susceptible to transient task-irrelevant emotional distraction than others.

### Approach, experimental design, and predictions

**Figure 1:**
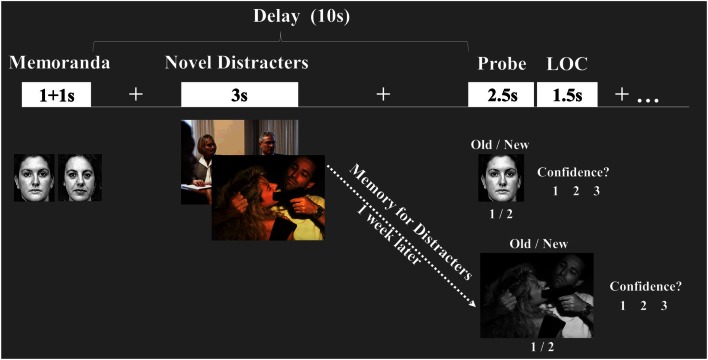
**Diagram of the combined WM-EM task**. Functional magnetic resonance imaging (fMRI) data were recorded while subjects performed a working memory (WM) task with distraction. WM performance was measured using an *Old/New* recognition memory task, followed by a level of confidence (LOC) task (1 = Low, 2 = Medium, 3 = High). EM for the distracters themselves was measured 1-week later, outside the scanner, and also involved *Old/New* and LOC assessments.

These issues were investigated using fMRI recording in conjunction with a novel experimental design that assessed both the EME and WMI effects of emotion, within the same participants. In a previous investigation, we examined similar issues by measuring the initial impact of emotional distraction on lower-level perceptual processing and the long-term EME effect (see Shafer and Dolcos, 2012 in the present research topic). Here, we investigated the link between these opposing effects by measuring the initial impact of emotional distraction on higher-level cognitive processes (i.e., WM), which may be differentially affected by distraction (Lavie, 2005). Specifically, we used an adapted version of our WM task with distraction (Dolcos and McCarthy, 2006), to assess not only the initial impact of emotional distraction on WM but also the long-term impact on EM for the distracters themselves (Figure 1). To investigate the role of individual differences, aspects of processing in both affective and cognitive domains where measured and investigated linked to differential emotional sensitivity and susceptibility to emotional distraction.

Based on the extant evidence concerning the enhancing and impairing effects of emotion discussed above, we made the following predictions. Regarding the first goal, we predicted that (1) if resource re-allocation by emotional distraction during the WM task coincides with the initiation of processing that also leads to enhanced EM for the distracters themselves, the same AMY regions should play a key role in both of these opposing effects. However, this would produce different effects in brain areas linked to initial WMI (reduced dlPFC activity) vs. long-term EME (increased MTL activity) effects, respectively; (2) coping with emotional distraction would be associated with increased activity in PFC regions. Regarding the second goal, we predicted that individual variations in the susceptibility to emotional distraction would differentially affect the response in emotion and cognitive/executive control brain areas. Specifically, participants with increased susceptibility to WMI would show (3) greater AMY increases and PFC reductions to emotional distraction, and (4) increased AMY activity and decreased PFC activity linked to measures indexing enhanced susceptibility to distraction and impaired executive control.

## Materials and Methods

### Subjects

Analyses were performed on data from 17 young (19–35 years of age) healthy right-handed female participants, recruited from Duke University community. We restricted our study to female participants in order to maintain homogeneity of the subject sample, as evidence shows that women and men differ in terms of general emotional reactivity (Shields, 1991; Lang et al., 1993; Hamann and Canli, 2004), response to emotional distraction (Iordan et al., 2013a), and emotion regulation (Thayer et al., 2003; Matud, 2004; McRae et al., 2008; Mak et al., 2009; Domes et al., 2010; Denkova et al., 2012). Also, this allowed a more direct comparison with findings from similar previous investigations (Dolcos and McCarthy, 2006; Dolcos et al., 2006, 2008). The experimental protocol was approved by the Institutional Review Board at Duke University Medical Center and all subjects provided informed consent.

### Experimental procedures

Subjects performed a combined WM-EM task measuring both the immediate impact of emotion on WM and the long-term impact on EM for the distracters themselves.

#### Working memory task

Subjects were scanned while performing a delayed-response WM task with novel distracters presented during the delay interval between memoranda and probes (Dolcos and McCarthy, 2006). The memoranda consisted of pairs of human faces (50% females/50% males) presented successively one at a time, that were masked to exclude non-facial features and displayed in black-and-white for increased task difficulty. The distracters consisted of highly arousing, negative emotional scenes (e.g., mutilations, aggressive behaviors) and low arousing neutral scenes (e.g., mundane activities), selected from the International Affective Picture System (IAPS, Lang et al., 2008) and supplemented with in-house pictures used in previous studies (Dolcos and McCarthy, 2006), to equate for complexity and human presence across conditions. The average IAPS arousal/valence ratings for the emotional and neutral scenes, respectively, were 5.91/2.32 and 3.53/5.32. To maximize their impact, distracters were presented in color.

Eight sets of 16 trials (8 emotional and 8 neutral distracters per set) were created and randomly assigned to 8 experimental blocks/runs. To avoid induction of longer-lasting effects, the trials within each run were pseudo-randomized, so that no more than two consecutive trials of the same type were presented. To prevent possible biases resulted from using the same run order, participants were assigned different run orders; a total of 8 different run orders were involved. Each trial started with the presentation of face memoranda (1 s + 1 s), which subjects were instructed to encode and maintain in WM during the delay interval (10 s) between the offset of the second memoranda and the onset of the memory probe. Presentation of the novel picture distracter started 4 s after the offset of the memoranda, and occurred for a total time of 3 s. Participants were instructed to look at the distracters but maintain focus on the WM task, and when the single face probe appeared they had to indicate by a button press whether the face was part of the current memorandum (*Old*) or not (*New*); 50% of the probes were *Old* and 50% were *New*. Subjects were instructed to make quick and accurate responses while the probes were on the screen, and then they also rated the level of confidence (LOC) of their responses, using a 3-point Likert scale (1 = *lowest*, 3 = *highest*). The LOC rating was followed by a 10 s inter-trial interval (ITI), to allow the hemodynamic response to return to baseline. The total length of each trial was 26 s.

#### Episodic memory task

One week following scanning, subjects performed a surprise memory task that tested EM for the emotional and neutral pictures previously presented as distracters during the WM task. The test included 192 pictures (96 emotional) out of which ~2/3 were old pictures. Old and new pictures did not reliably differ in normative intensity scores. All pictures were displayed in black-and-white for increased task difficulty. Trials within each block were pseudo-randomized, so that no more than two consecutive trials of the same type were presented, and participants were assigned different run orders. Each picture was displayed for 3 s and subjects had to indicate by a button press whether the picture was previously seen during the WM task (*Old*) or not (*New*). Participants were encouraged to make quick and accurate responses while the picture was on the screen, and then they also rated the LOC of their responses, using a 3-point Likert scale (2 s); the LOC rating was followed by a 2 s ITI.

### Additional behavioral and personality measures

These measures aimed at assessing aspects related to both emotion and executive processing. Following scanning, subjects rated the emotional intensity of the emotional and neutral distracters using a 9-point Likert scale (1 = *lowest*; 9 = *highest*). These ratings were assessed to confirm that the negative distracters were perceived as more emotional than the neutral distracters, and to calculate individual indices of emotional sensitivity to the distracters (see Behavioral Data Analyses). Given the possibility that differences in both emotional and cognitive control/executive processing can modulate the relationships between the immediate and long-term impact of emotion, measures indexing general emotion and executive processing were also assessed in participants. Aspects related to general emotional state were assessed using the Positive and Negative Affective Schedule (PANAS-S; Watson et al., 1988), both at the beginning and at the end of both parts of the study; repeated PANAS measures were involved to make sure that subjects’ emotional state did not dramatically change as a result of participating in the study. Aspects related to executive processing were assessed using the Barratt Impulsiveness Scale (BIS-11; Patton et al., 1995; Spinella, 2007), which measures a trait that has been consistently linked to impaired executive performance (Enticott et al., 2006; Pietrzak et al., 2008; Kam et al., 2012). This scale yields 3 second-order factors: attentional impulsiveness (AI), motor impulsiveness (MI), and non-planning impulsiveness (NpI).

### Imaging protocol

Scanning was conducted on a 4T GE scanner (General Electric, Milwaukee, WI, USA). After localizer, anatomical series, and high-order shimming, functional volumes were acquired using an inverse-spiral pulse sequence (echo time: 31 ms; field of view: 25.6 cm × 25.6 cm; repetition time: 2000 ms). Each volume consisted of 30 functional slices acquired axially (voxels size: 4 mm × 4 mm × 4 mm), thus allowing full-brain coverage. Anatomical scans consisted of high-resolution three-dimensional spin-echo structural images, which were acquired coplanar with the functional slices (1 mm × 1 mm × 1 mm; anatomical-functional ratio = 4:1).

### Behavioral data analyses

Responses in the WM task were classified in one of the four categories derived from signal detection theory (Macmillian and Creelman, 1991): (1) *Hits* = Probes from memoranda (*Old*) correctly classified as *Old*, (2) *Misses* = Probes from memoranda incorrectly classified as *New*, (3) *Correct Rejections* (CRs) = New probes correctly classified as *New*, and (4) *False Alarms* (FAs) = New probes incorrectly classified as *Old*. For the EM task, the probes were subsets of the distracters used in the WM task, supplemented with new pictures as foils. Responses in the EM task were classified similarly to the WM task into Hits, Misses, CRs, and FAs. Percentages of probes correctly identified as being *Old* or *New* were also calculated for each participant [% Correct = (% Hits + % CR)/2]. Although based on previous studies (Dolcos and McCarthy, 2006; Dolcos et al., 2008; Anticevic et al., 2010; Denkova et al., 2010) we expected that most participants would show impaired WM performance to emotional distraction, we also expected that this effect would not be consistent across all subjects (Dolcos et al., 2008). Hence, participants who showed the expected pattern of lower WM performance to emotional than to neutral distraction were assigned to the WMI subgroup, while the rest were assigned to the Non-WMI subgroup (see the Results for the average WM performance scores and the number of subjects in the WMI and Non-WMI subgroups). The dependent variables for the behavioral performance analyses were the percentage of correct scores in the WM and EM tasks. The independent variables for the same analyses were trial type (emotional vs. neutral) and subgroup (WMI vs. Non-WMI). Differences in performance between the two trial types (emotional vs. neutral) were assessed separately for the WM and EM tasks, using *t* statistics. Differences between the two subgroups (WMI vs. Non-WMI) were assessed using *t* statistics and mixed-model ANOVAs. Based on the subjects’ ratings of the emotional intensity of the distracters, individual indices of emotional sensitivity to the present distracters were calculated separately for each subject by subtracting the average of their own ratings for the neutral distracters from the average of their ratings for the emotional distracters. Finally, to assess the role of individual differences in the relationships between behavioral performance and personality, correlations between WM/EM performance and affective/executive measures were also calculated.

### fMRI data analyses

Imaging data analyses were performed using SPM2 in conjunction with in-house custom MATLAB scripts. Statistical analyses were preceded by the following preprocessing steps: quality assurance, TR alignment, motion correction, coregistration, normalization, and smoothing (8 mm^3^ Kernel). For individual analyses, task-related activity was identified by convolving a vector of the onset times of the distracters with a synthetic hemodynamic response and its temporal derivative. The general linear model, as implemented in SPM2, was used to model the effects of interests and other confounding effects (e.g., session effects and magnetic field drift). There were 14 first-level regressors: eight task variables (Emo *WM-R and EM-R*, Emo *WM-F and EM-R*, Emo *WM-R and EM-F*, Emo *WM-F and EM-F*, Neu *WM-R and EM-R*, Neu *WM-F and EM-R*, Neu *WM-R and EM-F*, Neu *WM-F and EM-F*) and six motion regressors (three translations, three rotations). Group analyses were conducted using random-effects models to assess the effect of distracter content. The following contrast images were taken to the second level: (1) contrasts linking WM impairment due to emotion and EM for the distracters themselves (i.e., Emo *WM-F and EM-R* > Neu *WM-F and EM-R*, and the reverse), (2) contrasts linking WM success in the face of emotional distraction and subsequent EM for the distracters themselves (i.e., Emo *WM-R and EM-R* > Neu *WM-R and EM-R*, and the reverse), (3) contrasts linking both WM and EM success, calculated separately for trials with emotional and neutral distracters that were subsequently remembered in the EM task (i.e., Emo *WM-R and EM-R* > Emo *WM-F and EM-R*, and Neu *WM-R and EM-R* > Neu *WM-F and EM-R*).

The main goals of fMRI data analyses were to (i) identify the neural mechanisms linking the immediate impact of emotional distraction on WM and the EM for the distracters themselves, and to (ii) investigate the role of individual differences in these effects. To accomplish these goals, brain regions in the ventral-affective and dorsal-executive neural systems, specifically sensitive to the presence of emotional distraction were defined as *a priori* regions of interest, based on our initial study using a similar WM task with distraction (Dolcos and McCarthy, 2006). That study involved three distracter conditions: emotional (Emo), neutral (Neu), and scrambled (Scr), and for the purpose of the current study the following two *t* maps were used: Emo > Scr, to identify regions of the ventral-affective system and Scr > Emo, to identify regions of the dorsal-executive system, both identified using an intensity threshold of *p* < 0.005, uncorrected (Lieberman and Cunningham, 2009). Activity within these *a priori* defined ROIs from Dolcos and McCarthy (2006) was further investigated to address the questions of the present investigation, as described below.

Related to our first main goal, to identify brain regions linking the WMI and EME effects of emotion, *t* maps contrasting the emotional and neutral distracters were computed for items that impaired WM and were later remembered: (Emo *WM-F and EM-R* > Neu *WM-F and EM-R*), for activity in the ventral-affective network and (Neu *WM-F and EM-R* > Emo *WM-F and EM-R*), for activity in the dorsal-executive network. To identify brain mechanism linking the initial effect on WM to the long-term impact on EM in conditions where participants coped with the presence of emotional distraction, we investigated activity for trials in which emotion enhanced EM without disrupting WM. For this, first, *t* maps contrasting the emotional and neutral distracters were computed for items that were associated with WM success and were later remembered: (Emo *WM-R and EM-R* > Neu *WM-R and EM-R*), for activity in regions of the ventral network, and (Neu *WM-R and EM-R* > Emo *WM-R and EM-R*), for activity in regions of the dorsal network. Then, to further check whether activity in these regions was also specifically linked to WM success, the maps identifying regions associated with WM success for items that were later remembered, as identified above, were inclusively masked with *t* maps contrasting activity for items associated with WM success vs. impairment. The latter were separately calculated for trials with emotional and neutral distracters that were subsequently remembered in the EM task: (Emo *WM-R and EM-R* > Emo *WM-F and EM-R*), for activity in ventral, and (Neu *WM-R and EM-R* > Neu *WM-F and EM-R*), for activity in dorsal regions. The main focus of the present investigation was on identifying the brain regions involved in *linking* the immediate impact of emotional distraction on WM and the subsequent EM for the distracters themselves. For this reason, the main analysis focused on trials corresponding to distracters meeting both criteria (impaired WM and were subsequently remembered: i.e., *WM-F and EM-R*). Activity for these trials was separately identified for the emotional and neutral distracters and then compared to each other. Hence, the link between WM and EM was identified at the level of the trials and the impact of emotion was calculated relative to the neutral stimuli with a similar outcome – i.e., Emo *WM-F and EM-*R > Neu *WM-F and EM-R*, for activity in the ventral system, and the reverse contrast for activity in the dorsal system. Importantly, the fact that some participants did not show an overall impairing effect of emotional distraction at the behavioral level did not affect the analyses of the fMRI data, as the trials linking the opposing effect of emotion on WM and EM could be identified in all participants regardless of the overall impact of emotional distraction on WM.

Related to the second main goal, the role of individual differences in the susceptibility to emotional distraction was investigated using two main analyses, as follows. One analysis involved comparisons of subjects showing a WMI effect (WMI subgroup) with those who did not (Non-WMI subgroup), in response to emotional relative to neutral distraction. This analysis involved a between-samples comparison, to identify differences in brain activity between these two subgroups, in the ventral and dorsal networks. For this, subject-level effects contrasting brain activity for emotional and neutral distracters were first calculated (i.e., Emo > Neu, in the ventral, and Neu > Emo, in the dorsal system), to be used as input for second level between-groups *t*-tests. Then, to make sure that regions identified by the between-groups analysis were also sensitive to the effects of emotional distraction, the resulting *t* maps were inclusively masked with statistical maps identifying a main effect of emotion in the targeted group (*in*creased vs. *de*creased activity in the ventral or dorsal systems, respectively). For example, increased activity to emotional distraction in the ventral system, in the WMI subgroup was identified by [WMI subgroup (Emo > Neu) > Non-WMI subgroup (Emo > Neu)] ∩ [WMI subgroup (Emo > Neu)]. This more stringent approach ensured that the effect captured by the between-groups comparison came from a difference going in the expected direction in the group of interest, and is not driven by the lack of effects coupled with differences going in opposite direction in the other group. The other main analysis involved identification of brain-behavior relationships by calculating co-variations between the fMRI signal and behavioral and personality measures, to further clarify the significance of effects in brain areas showing differences in activation. The focus was on measures indexing susceptibility to distraction, as reflected in the WM performance and personality scales, such as BIS, which measures a personality trait that has been linked to impaired executive performance. While the stringent masking criteria employed may have offered enough protection against Type I error, given the relatively small sample (Yarkoni, 2009), the findings regarding individual differences are provisional in nature and should be treated with caution.

Within the *a priori* defined *ventral* and *dorsal* ROIs (based on the Dolcos and McCarthy, 2006 data identified at a threshold of *p* < 0.005), activity in the areas identified by the contrasts described above was assessed with a threshold of *p* < 0.05, uncorrected. This allows direct comparison with a complementary study investigating similar issues in the perceptual domain (Shafer and Dolcos, 2012). Unless otherwise noted, an extent threshold of five contiguous voxels was used in all analyses. Finally, in-house manually traced ROIs on the normalized SPM brain template and the Automated Anatomical Labeling (AAL) toolbox (Tzourio-Mazoyer et al., 2002) were used to confirm localization of and to display the effects from AMY and HC.

## Results

### Behavioral results

Overall, WM performance was equivalent across both trial types [emotion = 74.8%, neutral = 74.8%; *t*(16) = 0.01; *p* = 0.989]. Although this result was inconsistent with the expected pattern of lower WM performance in response to emotional relative to neutral distracters observed in other previous investigations using similar tasks with emotional distraction (e.g., Dolcos and McCarthy, 2006; Anticevic et al., 2010; Denkova et al., 2010), this result was not totally surprising given the expected individual variation in the response to emotional distraction (Dolcos et al., 2008). To investigate whether this null finding at the group level was related to individual differences in behavioral responses, we examined whether subsets of subjects showed different WM performance to emotional relative to neutral distracters. For this, the subjects sample was split into two subgroups, as a function of WM performance, as follows. Subjects showing the expected pattern of impaired WM performance to emotional relative to neutral distracters (WMI subgroup), and subjects not showing this pattern (Non-WMI subgroup). About 60% of the subjects (*N* = 10) showed the pattern of impaired WM performance to emotional relative to neutral distracters [WMI subgroup: emotional = 75%, neutral = 79%; *t*(9) = 2.76, *p* = 0.022], whereas the remaining ~40% of the subjects (*N* = 7) did not show it (Non-WMI subgroup: emotional = 74.4%, neutral = 68.7%). It should be noted that the fact that some participants did not show an overall impairing effect does not affect the analyses of the fMRI data focusing on the trials associated with WM errors and successful subsequent EM. Regarding the EM performance, as expected, the majority of subjects (~90%, *N* = 15) remembered better the emotional relative to neutral distracters [emotional = 75.5%, neutral = 69.3%; *t*(16) = 3.44, *p* = 0.003], and this effect was strongest in trials associated with the highest level of confidence [LOC3: emotional = 35.1%, neutral = 27.9%; *t*(16) = 3.52, *p* = 0.003]. Further explorations of EM performance revealed a tendency for the participants who showed impaired WM performance to emotional distraction (WMI subgroup) to have better EM for the distracters themselves (WMI subgroup: emotional EM = 78.1%, neutral EM = 72.8%; Non-WMI subgroup: emotional EM = 71.8%, neutral EM = 64.4%), although this difference was not statistically significant. A mixed-design ANOVA (WM subgroup × Distracter type) on EM performance confirmed these impressions, yielding a significant main effect of Distracter type [*F*(1, 15) = 11.62, *p* = 0.004], a marginal effect of subgroup [(*F*(1, 15) = 4.18, *p* = 0.059], and no interaction effects (*p* = 0.585). Overall, these results show a differential impact of emotional distraction on WM vs. EM and suggest a link between the initial and long-term effects of distraction.

To further elucidate these differential effects of emotional distraction on WM and EM, additional analyses were performed on behavioral and personality data. These analyses revealed that subjects showing systematic impaired WM performance in the presence of emotional distraction (WMI subgroup) also experienced the emotional distracters as more emotional. Specifically, in addition to overall greater ratings for emotional than neutral distracters observed across all subjects [emotional = 6.5; neutral = 2.5; *t*(16) = 15.56, *p* < 0.001], the WMI subgroup also perceived the negative pictures as more negative relative to the neutral pictures (WMI subgroup: emotional = 6.8, neutral = 2.5; Non-WMI subgroup: emotional = 6, neutral = 2.6). This was reflected in higher individual indices of emotional sensitivity [*t*(15) = 1.77, *p* = 0.049, one-tailed]. In addition, the WMI subgroup also had lower scores in the Self Control subscale of the BIS questionnaire [*t*(15) = 1.77, *p* = 0.049, one-tailed]. There were no other differences between the WMI vs. Non-WMI subgroups. Interestingly, correlation analyses showed that the scores for negative general affective state, as assessed by post-WM task PANAS-state, were negatively correlated* with the WM scores for trials with negative distracters (*r* = −0.51, *p* = 0.043; *based on data from 16 subjects due to missing PANAS values for one participant). In other words, participants who were affected more by the negative distraction during the WM task also reported more negative emotions following the task.

Taken together, the behavioral results identified differential effects of emotion on WM vs. EM, consistent with a link between the initial and long-term effects of distraction, and that these effects were influenced by individual differences. Results from analyses of brain imaging data conducted to investigate the neural correlates of these effects are presented below.

### fMRI results

#### Neural mechanisms linking the differential impact of emotional distraction on WM and EM

##### Concomitant WMI and EME effects of emotion were associated with increased AMY-HC activity and reduced dlPFC activity

**Figure 2:**
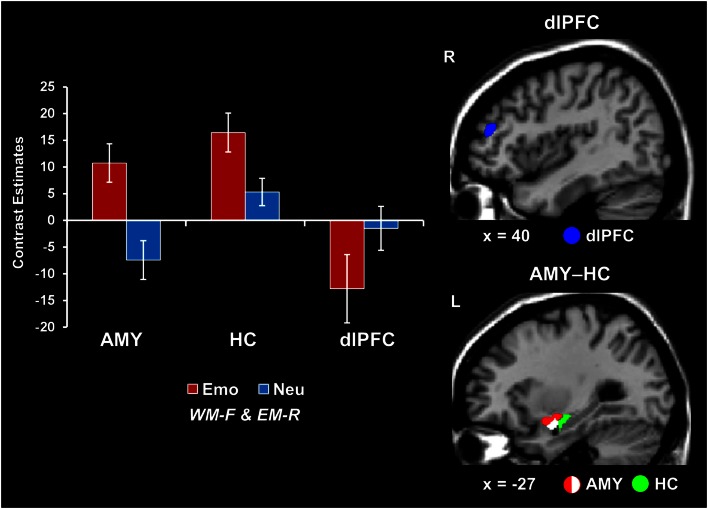
**Opposing patterns of activity in AMY and HC vs. PFC linked to WMI and EME effects of emotion**. Increased activity in both AMY (red blob) and HC (green blob) and greater deactivation in the dlPFC (MFG, BA46; blue blob) were observed in response to emotional relative to neutral distracters that impaired WM performance and were later remembered (i.e., Emo *WM-F and EM-R* > Neu *WM-F and EM-R*, for activity in the ventral and Neu *WM-F and EM-R* > Emo *WM-F and EM-R* for activity in the dorsal systems). A negative correlation was also identified between left AMY activity and WM performance to emotional distracters (white blob; see Results). The bar graphs show the contrast estimates, as extracted from for peak voxels in left AMY (Talairach coordinates: *x* = −27, *y* = 3, *z* = −10), HC (Talairach coordinates: *x* = −28, *y* = −12, *z* = −12), and the right dlPFC (MFG, BA46; Talairach coordinates: *x* = 43, *y* = 38, *z* = 23). The activation maps are superimposed on high-resolution brain images displayed in sagittal view (*x* indicates the Talairach coordinate on the left-right axis of the brain). AMY, amygdala; HC, hippocampus; dlPFC, dorsolateral prefrontal cortex; MFG, middle frontal gyrus; Emo/Neu *WM-F and EM-R*, emotional/neutral distracters that impaired WM and were later remembered. Error bars represent standard errors of means.

Analyses contrasting activity for the emotional and neutral distracters that disrupted face WM performance but were later remembered in the EM task (i.e., Emo *WM-F and EM-R* > Neu *WM-F and EM-R*) identified increased activity in the same AMY region linked to both WMI and EME effects (see red blob in Figure 2). However, the same trials were associated with opposing modulation of HC (increased) and dlPFC (decreased; as identified by the reverse contrast Neu *WM-F and EM-R* > Emo *WM-F and EM-R*) activity (see the green and blue blobs in Figure 2 depicting HC and dlPFC, respectively; see also Table 1). In addition, investigation of brain-behavior relationships linked to differences in WM performance identified a negative correlation between left AMY activity to emotional vs. neutral distracters, and WM performance to emotional distracters (*r* = −0.55, *p* = 0.01; Talairach coordinates: *x* = −23, *y* = −4, *z* = −14; see the white blob overlapping with the AMY region illustrated by the red blob in Figure 2). In other words, participants who showed increased AMY activity to emotional distracters were also more impaired in WM performance by the presence of emotional distraction. Overall, consistent with a bottom-up effect of emotional distraction, increased AMY activity in the presence of emotional distraction was associated with lower WM performance and increased EM.

**Table 1:** **Opposing effects in ventral affective and dorsal-executive neural systems linked to WMI and EME effects of emotion**.

Brain regions		BA	Talairach coordinates			*T* value	Mask
	
			***x***	***y***	***z***		
**Emo WM-F and EM-R > Neu WM-F and EM-R**							
mPFC	L medial frontal gyrus	8	−16	32	40	2.77	4.18
	L superior frontal gyrus	8/9	−16	31	50	2.79	4.06
	R medial frontal gyrus	8	6	44	41	5.15	4.83
	R superior frontal gyrus	9	14	49	20	5.12	5.33
vlPFC	L inferior frontal gyrus	45	−49	28	10	2.52	3.77
	R inferior frontal gyrus	44/45	54	12	17	2.55	3.33
latPFC	R inferior frontal gyrus	9	51	19	21	2.97	5.52
MFC	R superior frontal gyrus	6	6	27	54	2.79	3.41
PrCG	R precentral gyrus	6	43	−1	34	3.24	3.71
TOC	L fusiform gyrus	37	−45	−52	−16	3.45	9.62
	L middle temporal gyrus	37/39	−46	−58	5	3.54	6.51
	L inferior temporal gyrus	19	−46	−57	−6	4.48	6.71
	L middle occipital gyrus	19	−46	−77	11	3.03	7.06
	R middle temporal gyrus	19	36	−59	14	3.73	3.38
	R middle occipital gyrus	18	28	−81	8	4.31	9.97
	R lingual gyrus	18	36	−65	−5	2.95	5.1
Precuneus	R precuneus	7	24	−55	46	6.8	4.03
	R precuneus	31	28	−74	16	5.55	6.71
	L precuneus	31	−27	−75	18	2.01	4.22
MTL	L amygdala		−27	3	−10	7.88	4.6
	L hippocampus		−27	−12	−8	3.24	5.27
	R amygdala		18	−5	−7	6.53	5.11
	R hippocampus		32	−8	−10	2.48	4.75
Insula	L insula		−34	−3	14	2.73	3.3
Subcortical	L hypothalamus		−8	−5	−4	3.34	4.67
	L lateral globus pallidus		−23	−8	−4	3.69	3.93
	L medial globus pallidus		−16	−5	−4	3.3	3.85
	L thalamus		−12	−17	6	2.68	4.64
	R medial globus pallidus		18	−5	−3	7.57	3.42
	R putamen		21	6	−2	3.96	3.38
	R thalamus		6	−18	14	2.33	3.09
Midbrain	L red nucleus		−1	−27	−6	5.06	5.23
	R mammillary body		7	−8	−7	4.58	4.59
Cerebellum	L declive		−45	−67	−17	3.25	12.42
**Neu WM-F and EM-R > Emo WM-F and EM-R**							
dlPFC	R middle frontal gyrus	46	43	38	23	2.82	3.94
MFC	R medial frontal gyrus	6	6	−18	57	2.14	3.53
PrCG	R precentral gyrus	6	51	−7	19	2.82	3.1
PCL	L paracentral lobule	6	−9	−30	59	3.39	3.67
	R paracentral lobule	6	6	−30	59	2.2	2.99
LTC	L superior temporal gyrus	22	−57	−9	6	5.38	2.98
	L superior temporal gyrus	41	−49	−36	11	5.13	4.87
	L middle temporal gyrus	21	−60	−35	0	2.46	3.08
	L middle temporal gyrus	20	−57	−38	−7	2.25	3.19
	R superior temporal gyrus	22	54	−24	3	5.39	3.35
	R superior temporal gyrus	41/42	51	−33	13	4.29	3.22
SPL	R superior parietal lobule	7	32	−73	44	2.6	3.35
TPC	L angular gyrus	39	−50	−68	33	2.13	4.26
	L inferior parietal lobule	39	−50	−62	41	2.25	5.19
Cuneus	L cuneus	19	−5	−76	37	3.64	3.54
	L cuneus	18	−16	−99	2	3.28	3.61
PHC	L parahippocampal gyrus	19	−31	−46	−1	2.55	3.48
	R parahippocampal gyrus	19	32	−43	−3	2.45	4.7
Subcortical	L caudate		−16	23	17	3.56	3.44

The table identifies brain regions mediating both the WM impairing and EM enhancing effects of emotion by contrasting emotional (Emo) and neutral (Neu) items that impaired WM and were later remembered (*WM-F and EM-R*). Effects in the ventral affective (Emo *WM-F and EM-R* > Neu *WM-F and EM-R*) and dorsal executive (Neu *WM-F and EM-R* > Emo *WM-F and EM-R*) networks were masked by their corresponding *a priori* ROIs (i.e., Emo > Scr in the ventral and Scr > Emo in the dorsal networks, respectively; see Materials and Methods). mPFC, medial prefrontal cortex; vlPFC, ventrolateral prefrontal cortex; latPFC, lateral prefrontal cortex; MFC, medial frontal cortex; PrCG, precentral gyrus; TOC, temporal-occipital cortex; MTL, medial-temporal lobe; dlPFC, dorsolateral prefrontal cortex; PCL, paracentral lobule; LTC, lateral temporal cortex; SPL, superior parietal lobule; TPC, temporal-parietal cortex; PHC, parahippocampal cortex. Significance thresholds are *p* < 0.05 for the effects of emotion and *p* < 0.005 for the *a priori* masks.

##### Increased vlPFC activity was linked to coping with emotional distraction and enhanced EM performance

**Figure 3:**
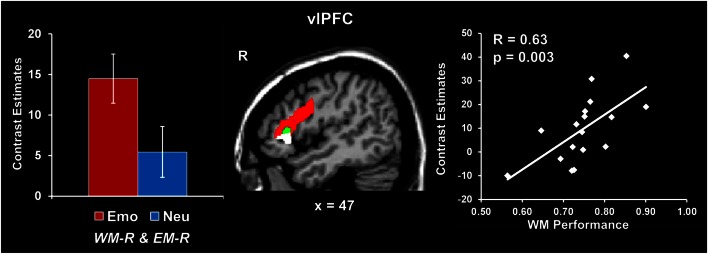
**Increased right vlPFC activity linked to coping with emotional distraction and enhanced EM**. Right vlPFC showed increased activity to emotional distracters associated with WM success and later remembered relative to both neutral distracters associated with WM success (red blob) and emotional distracters associated with WM impairment (green blob). A positive correlation was also identified between activity in this right vlPFC area and WM performance for emotional distraction (white blob; see Results). In contrast, the left inferior frontal cortex showed increased activity to emotional distracters associated with WM success relative to emotional distracters associated with WM impairment, independent of whether they were later remembered or not (Talairach coordinates: *x* = −46, *y* = 8, *z* = 22; not shown). This suggests a hemispheric dissociation between brain activity involved in coping with emotional distraction (left vlPFC) and linking coping mechanisms with increased subsequent EM for the distracting information (right vlPFC). The bar graph shows contrast estimates for the peak voxel in right vlPFC for the comparison between emotional and neutral stimuli associated with WM success and later remembered (Talairach coordinates: *x* = 43, *y* = 23, *z* = 14). The scatter plot shows the co-variation between brain activity and WM performance, as extracted from the peak voxel of the green blob (Talairach coordinates: *x* = 47, *y* = 24, *z* = 7). The activation maps are superimposed on a high-resolution brain image displayed in sagittal view (*x* indicates the Talairach coordinate on the left-right axis of the brain). vlPFC, ventrolateral PFC; Emo/Neu *WM-R and EM-R*, emotional/neutral distracters that did not impair WM and were later remembered. Error bars represent standard errors of means.

Analyses contrasting activity for the emotional and neutral distracters that did not impair WM performance but were later remembered (i.e., Emo *WM-R and EM-R* > Neu *WM-R and EM-R*) identified increased activity in a right vlPFC region (red blob in Figure 3; see also Table 2). Importantly, activity in this vlPFC region overlapped with areas associated with successful coping with emotional distraction, as revealed by greater activity to emotional distracters associated with WM success than to those that impaired WM (i.e., Emo *WM-R and EM-R* > Emo *WM-F and EM-R*; see green blob in Figure 3). Moreover, investigation of brain-behavior relationships showed a positive correlation between activity in these right vlPFC areas, in response to emotional vs. neutral distracters associated with WM success and later remembered, and WM performance for emotional distracters (*r* = 0.63, *p* = 0.003; Talairach coordinates: *x* = 47, *y* = 24, *z* = 7; see the white blob in Figure 3). Specifically, consistent with a role of this region in coping with emotional distraction, participants who showed increased vlPFC activity also had higher WM performance in the presence of emotional distraction.

**Table 2:** **Differential effects in the ventral and dorsal neural systems linked to successful coping with emotional distraction and enhanced EM for the distracters themselves**.

Brain regions		BA	Talairach coordinates			*T* value	Mask
	
			*x*	*y*	*z*		
**Emo WM-R and EM-R > Neu WM-R and EM-R**							
mPFC	L superior frontal gyrus	8	−9	43	48	3.24	5.23
	L medial frontal gyrus	9	−1	44	34	3.53	7.69
	R superior Frontal Gyrus	9	14	49	20	3.33	5.33
vlPFC	L inferior frontal gyrus	47	−34	26	−5	3.32	3.29
	R inferior frontal gyrus	45	43	23	14	5.38	5.25
latPFC	R inferior frontal gyrus	9	47	7	24	2.91	5.38
	R inferior frontal gyrus	44	54	16	17	2.6	4.51
PrCG	R precentral gyrus	6	43	−4	30	4.17	3.21
TP	L superior temporal gyrus	38	−38	0	−14	4.18	3.21
TOC	L inferior temporal gyrus	37	−49	−65	−3	5.73	9.52
	L fusiform gyrus	37/19	−49	−49	−16	4.62	8.03
	R fusiform gyrus	19/37	40	−65	−8	3.11	7.59
Precuneus	R precuneus	7/19	17	−66	42	4.74	3.46
Cuneus	R cuneus	18	24	−79	19	2.99	7.79
LOC	R middle occipital gyrus	19	36	−78	15	3.5	4.97
MTL	L amygdala		−30	0	−14	3.61	5.75
	R amygdala		29	−1	−6	3.75	5.5
Subcortical	L caudate		−12	1	15	3.19	4.2
	L thalamus		−5	−32	1	5.68	3.13
	L hypothalamus		−5	−5	−3	3.27	5.5
	L lateral globus pallidus		−19	−5	−4	3.11	3.57
	R thalamus		3	−28	2	5.6	3.06
	R medial globus pallidus		18	−5	−7	2.76	6.11
	R claustrum		36	3	−6	4.84	3.29
Midbrain	L mammillary body		−1	−12	−4	3.51	3.77
	R mammillary body		7	−8	−7	2.87	4.59
Cerebellum	L culmen		−42	−48	−23	3.89	11.81
	R culmen		32	−52	−18	2.91	10.6
**Neu WM-R EM-R > Emo WM-R EM-R**							
rPFC	L superior frontal gyrus	10	−31	50	16	3.54	5.12
	L middle frontal gyrus	10	−34	43	4	3.65	5.54
	R superior frontal gyrus	10	32	50	13	3.25	4.51
dlPFC	L middle frontal gyrus	9	−35	26	28	2.59	3.54
	R middle frontal gyrus	9	39	33	37	4	3.37
latPFC	L middle frontal gyrus	8	−35	25	42	2.69	3.05
LFC	R middle frontal gyrus	6	32	8	52	3.93	3.06
PrCG	R precentral gyrus	6	54	−6	8	6.11	3.51
	L precentral gyrus	9	−38	14	37	2.58	4.12
PoCG	R postcentral gyrus	4	13	−38	62	2.36	3.02
PCL	R paracentral lobule	6	6	−30	59	2.22	2.99
IPL	R inferior parietal lobule	40	46	−58	39	3.06	7.62
STC	L superior temporal gyrus	22	−49	−36	7	3.51	3.32
	L middle temporal gyrus	22/21	−57	−39	7	2.89	3.16
	R superior temporal gyrus	41	58	−25	10	4.27	3.62
	R superior temporal gyrus	13	47	−18	11	3.67	3.33
	R transverse temporal gyrus	41	43	−33	13	3.02	3.9
TPC	L angular gyrus	39	−35	−72	33	3.64	3.28
	L supramarginal gyrus	40	−50	−53	34	6.56	6.09
PCC	L posterior cingulate	23	−5	−30	27	2.85	4.69
	R cingulate gyrus	23	2	−34	26	2.91	3.46
Precuneus	L precuneus	39	−39	−65	37	3.1	3.32
	R precuneus	19	39	−73	34	3.61	6
Cuneus	L cuneus	17/18	−12	−99	−2	2.79	3.52
Cerebellum	L dentate		−19	−55	−27	2.88	3.04

The table identifies brain regions mediating both successful coping with emotional distraction and EM enhancing effects of emotion by contrasting emotional (Emo) and neutral (Neu) items that were associated with WM success and were later remembered (*WM-R and EM-R*). Effects in the ventral affective (Emo *WM-R and EM-R* > Neu *WM-R and EM-R*) and dorsal executive (Neu *WM-R and EM-R* > Emo *WM-R and EM-R*) networks were masked by their corresponding *a priori* ROIs (i.e., Emo > Scr in the ventral and Scr > Emo in the dorsal networks, respectively; see Materials and Methods). mPFC, medial prefrontal cortex; vlPFC, ventrolateral prefrontal cortex; latPFC, lateral prefrontal cortex; PrCG, precentral gyrus; TP, temporal pole; TOC, temporal-occipital cortex; LOC, lateral occipital cortex; MTL, medial-temporal lobe; rPFC, rostral prefrontal cortex; dlPFC, dorsolateral prefrontal cortex; LFC, lateral frontal cortex; PoCG, postcentral gyrus; PCL, paracentral lobule; IPL, inferior parietal lobule; STC, superior temporal cortex; TPC, temporal-parietal cortex; PCC, posterior cingulate cortex. Significance thresholds are *p* < 0.05 for the effects of emotion and *p* < 0.005 for the *a priori* masks.

#### The role of individual differences in the impact of emotional distraction

##### Participants who were more susceptible to WMI by emotional distraction showed greater amygdala increases and PFC reductions

**Figure 4:**
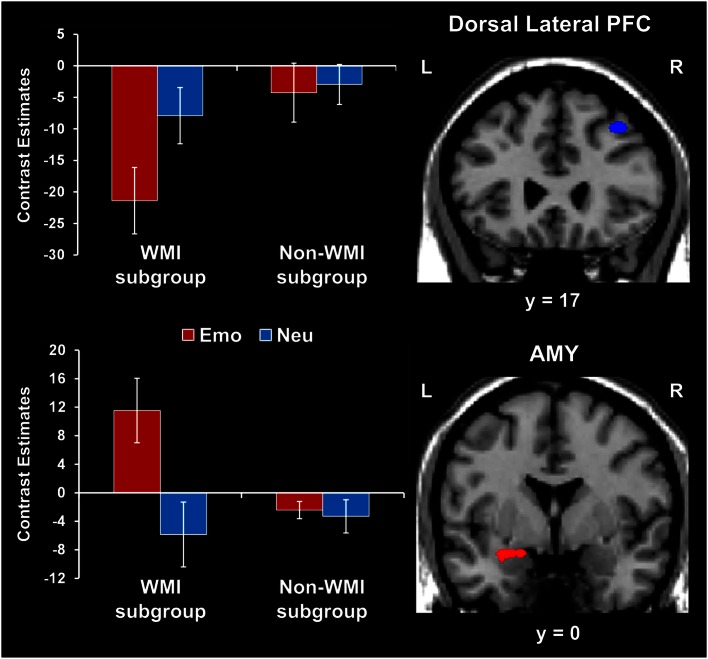
**Opposing effects of individual differences in susceptibility to emotional distraction on AMY and dorsal lateral PFC**. Participants who were more susceptible to WMI by emotional distraction (WMI subgroup) showed greater AMY increases (red blob) and dorsal lateral PFC reductions (blue blob), relative to the Non-WMI subgroup. The bar graph shows contrast estimates for the peak voxels in AMY (Talairach coordinates: *x* = −27, *y* = 3, *z* = −10) and dorsal lateral PFC (Talairach coordinates: *x* = 36, *y* = 17, *z* = 46), for the two subgroups. The activation maps are superimposed on a high-resolution brain image displayed in coronal view* (*y* indicates the Talairach coordinate on the anterior-posterior axis of the brain). *Three voxels overlapping with the mask were identified in the dorsal lateral PFC at this location. AMY, amygdala; PFC, prefrontal cortex; Error bars represent standard errors of means.

Consistent with the behavioral results, fMRI analyses comparing brain activity between participants showing impaired WM performance compared to those who were not impaired by emotional distraction (WMI subgroup vs. Non-WMI subgroup) identified increased AMY activation and dlPFC *de*activation in the WMI subgroup (see the red and blue blobs in Figure 4 depicting AMY and dlPFC areas showing differences in activation and, respectively, *de*activation to emotional vs. neutral distraction, between the WMI and Non-WMI subgroups). Thus, individual differences in the susceptibility to emotional distraction were associated with opposing effects in ventral (AMY) and dorsal (dlPFC) regions.

##### Amygdala activity increased and dlPFC activity decreased with measures of impulsivity

**Figure 5:**
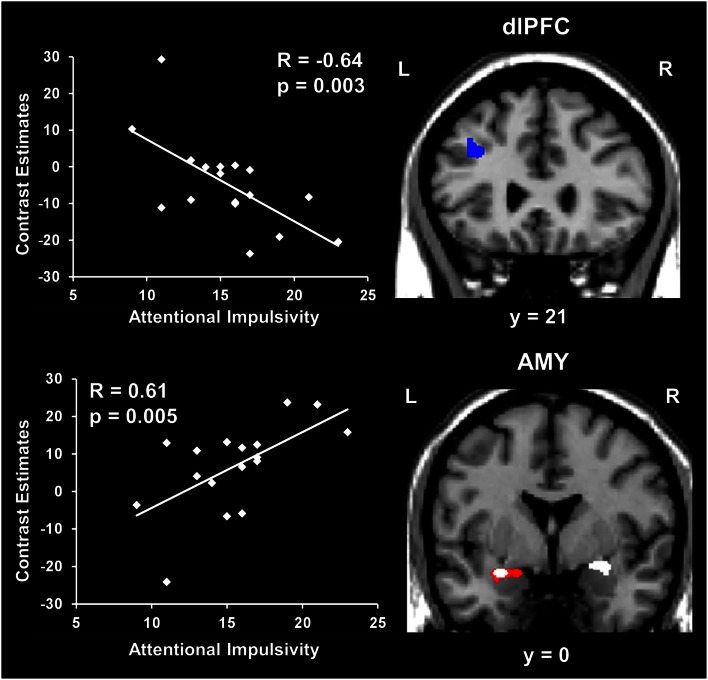
**Opposing co-variation of AMY and dlPFC activity to emotional distraction with individual differences in attentional impulsivity**. Bilateral AMY activity increased (white blobs) and left dlPFC activity (BA 8/9) decreased (blue blob) with individual scores of attentional impulsivity (AI). The positive correlation identified in the left AMY (white blob) overlapped with the AMY area showing difference in activation to emotional vs. neutral distraction illustrated in Figure 4 (red blob). The scatter plots illustrate the co-variation between brain activity for emotional vs. neutral distracters and AI scores. Contrast estimates in the scatter plots are extracted from the peak voxels in right AMY (Talairach coordinates: *x* = 25, *y* = −1, *z* = −6) and left dlPFC (Talairach coordinates: *x* = −35, *y* = 21, *z* = 38). The activation maps are superimposed on a high-resolution brain image displayed in coronal view (*y* indicates the Talairach coordinates on the anterior-posterior axis of the brain). AMY, amygdala; dlPFC, dorsolateral prefrontal cortex. Error bars represent standard errors of means.

Exploratory analyses were also performed to investigate possible relationships between individual differences in personality measures indexing impaired executive control (i.e., impulsivity) and brain activity. These analyses also targeted ventral affective and dorsal-executive regions showing sensitivity to emotional distraction, and where differences in activity were observed between the WMI and Non-WMI subgroups. These analyses revealed opposing relationships between brain activity in response to emotional distraction and individual scores for the AI subscale of BIS, in AMY and dlPFC (Figure 5). Specifically, AMY activity showed a positive correlation (see white blobs in Figure 5), whereas dlPFC activity showed a negative correlation (see blue blob in Figure 5) with the AI scores. In other words, participants with higher AI scores showed increased activity to emotional distraction in basic ventral emotion processing regions (AMY) and concomitant reduced activity in dorsal-executive regions (dlPFC). Notably, the positive correlation in the left AMY overlapped with the AMY area illustrated in Figure 4, as showing greater response to emotional distraction in the WMI subgroup (see the white blob overlapping with the red blob in Figure 5). Also, the positive correlation in the right AMY illustrated in Figure 5 was driven by the WMI subgroup (WMI subgroup: *r* = 0.74, *p* = 0.008; Non-WMI subgroup: *r* = 0.07, *p* = 0.44). Thus, individual differences in attentional impulsivity were associated with opposing patterns of co-variation with activity in ventral (AMY) and dorsal (dlPFC) regions.

## Discussion

The present study used a novel combined WM-EM experimental paradigm to investigate (i) the relationship between the immediate impact of emotional distraction on WM and the long-term impact on EM, and (ii) the role of individual differences in the impact of emotional distraction. The first goal yielded two main findings: (1) emotional pictures that disrupted face WM but enhanced subsequent picture EM were associated with increased AMY and HC activity coupled with reduced dlPFC activity (Figure 2); (2) trials in which emotion enhanced EM without disrupting WM were associated with increased ventrolateral PFC activity (Figure 3). The second goal yielded two additional findings: (3) participants who were more susceptible to the WMI effect of emotion showed greater AMY increases and PFC reductions (Figure 4); (4) AMY activity increased and dlPFC activity decreased with measures of attentional impulsivity (Figure 5). These findings are discussed in turn below.

### The link between opposing effects of emotional distraction on WM and EM

(1) The present results support the idea that the relationship between the immediate impact of emotion on WM and the long-term enhancement of EM is modulated by both direct/bottom-up MTL-based and mediated/top-down PFC-based mechanisms, and that AMY has a central role in both effects. Analyses contrasting the emotional and neutral distracters that impaired WM performance and were later remembered showed that the same AMY region was linked to both of these opposing effects. However, the same trials were associated with opposing modulation of HC (increased) and dlPFC (decreased) activity. These results are consistent with previous investigations linking AMY-HC engagement to a direct route contributing to the memory-enhancing effect of emotion (Dolcos et al., 2004a,b, 2011, 2012; Kensinger and Corkin, 2004) and with studies showing that emotional distraction is linked to increased activity in ventral-affective regions (e.g., AMY) and greater *de*activation in dorsal-executive regions (e.g., dlPFC) (Dolcos and McCarthy, 2006; Dolcos et al., 2006, 2008; Anticevic et al., 2010; Chuah et al., 2010; Denkova et al., 2010).

The fact that in the present study these effects were observed within the same participants provides strong evidence that reallocation of processing resources by emotional distraction during the WM task is one of the mechanisms that contribute to better memory for the distracters themselves. Specifically, possibly as a result of activating mechanisms signaling potential danger, processing of task-irrelevant negative distraction diverts processing resources from the main WM task to processing emotional distracters, which may lead to dlPFC *de*activation (Dolcos and McCarthy, 2006), while simultaneously initiating processing that leads to enhanced EM for the distracting stimuli, via a MTL-dependent route. Alternatively, it is possible that deactivation in some of the dorsal brain areas may reflect reduced executive control to focus on the WM task, possibly due to engagement in other operations that help reduce the impact of emotional distraction; this matter should be addressed in future investigations. In addition, the negative co-variation between left AMY activity and WM performance to emotional distracters is consistent with a bottom-up effect of emotional distraction, in which increased AMY activity in the presence of emotional distraction is associated with lower WM performance and increased EM, thus pointing to a role of bottom-up/MTL-based mechanisms in these effects.

These results are consistent with findings from another investigation from our group (Shafer and Dolcos, 2012), which also identified AMY-HC activity as being common to both the immediate/impairing and the long-term/enhancing impact of emotion, but in the context of a lower-level perceptual task, and under conditions of limited processing resources available at encoding. This suggests that emotional distracters which initially impair cognitive performance, either in the context of lower/perceptual or higher/WM processing level, engage similar bottom-up/direct AMY-MTL-based mechanisms that allow them to be better remembered later. However, as discussed in the next section, unlike our previous investigation, the present study also identified top-down/indirect mechanisms contributing to the EME effect of emotion, which were linked to initial coping with emotional distraction.

(2) Turning to our second main findings, increased vlPFC activity for emotional distracters associated with stimuli that did not impair WM but were later remembered provides evidence linking the mechanisms involved in coping with emotional distraction with those involved in enhanced EM. Increased vlPFC activity and AMY-vlPFC coupling have been linked to the engagement of PFC control mechanisms in order to cope with distracting emotions, leading to a diminution of the negative impact of distracters on on-going cognitive processes (Dolcos et al., 2006; Chuah et al., 2010; Denkova et al., 2010). Moreover, there is also evidence that deployment of coping/emotion regulation strategies modulates EM for emotional stimuli (Richards and Gross, 1999, 2000; Bonanno et al., 2004; Dillon et al., 2007), with some strategies leading to enhanced emotional memory (Dillon et al., 2007). This suggest that the initial engagement of emotion regulation strategies to cope with emotional distraction during WM in the present study also contributed to subsequent better EM for the distracters themselves, probably due to increased strategic influences on stimulus elaboration linked to a deeper level of processing (Dillon et al., 2007).

It should be noted that although overlapping areas of the right vlPFC also showed increased activity in response to emotional relative to neutral distracters that impaired WM performance and were later remembered (Table 1), it also showed greater activity to emotional distracters associated with WM success than to those that impaired WM performance (Figure 3). Thus, together with the pattern of positive co-variation in this brain area, linking increased right vlPFC activity with better WM performance to emotional distraction, these results are consistent with the idea that enhanced recruitment of this area is associated with successful coping with emotional distraction. On the other hand, the observed increased right vlPFC activity for WM trials showing impairment in the presence of emotional distracters that were subsequently remembered was probably reflective of unsuccessful engagement mechanisms to cope with emotional distraction, that yet also contributed to enhanced memory for the distracters themselves. Overall, the present findings show that activity in specific areas of the right vlPFC reflects the deployment of control mechanisms engaged to cope with emotional distraction and reduce the WMI effect, which also has an indirect/mediated contribution to the EME effect.

Together, the findings regarding the neural correlates linking the WMI and EME effects of emotion suggest that the same bottom-up mechanisms, involving the AMY and HC, contribute to both WMI and EME effects of emotion, and that specific top-down mechanisms, involving the right vlPFC, contribute to coping with emotional distraction and to the EME effect of emotion.

### The role of individual differences in the impact of emotional distraction

(3) The present findings expand previous evidence suggesting individual variation in the response to emotional distraction (Dolcos et al., 2008) by providing insight into the factors that may influence this phenomenon and the associated neural correlates. Regarding the factors influencing differential susceptibility to emotional distraction, participants whose WM performance was impacted by emotional distraction (i.e., the WMI subgroup) also rated the negative distracters as more negative and had lower scores in a measure indexing executive control (BIS-Self Control); also, overall, subjects who had lower WM performance in the presence of emotional distraction also experienced higher negative affect following the WM task. Moreover, consistent with the idea that individual differences in the initial response to emotional distraction may also influence its long-term impact, it is possible that increased overall EM for the distracters themselves in the WMI subgroup may be linked to reallocation of resources during the initial processing of task-irrelevant information, which in turn led to a long-term EM boost for the distracting information. Regarding the neural correlates, consistent with the behavioral results, fMRI results showed that participants who were more susceptible to the WMI effect of emotion showed greater increases in ventral/bottom-up regions (AMY) and greater reductions in top-down regions (dlPFC). These findings provide novel evidence concerning the neural correlates of increased susceptibility to distracting emotions, and complement previous investigations pointing to the role of individual differences in the response to emotional distraction (Dolcos et al., 2008; Denkova et al., 2010; Iordan et al., 2013a).

(4) Providing further support to the differences in activation, correlation analyses revealed opposing patterns of co-variation of AMY and dlPFC activity with measures of trait AI – i.e., AMY activity increased and dlPFC activity decreased with AI scores. Interestingly, the group-level positive correlation with AI scores in the right AMY was driven by the subjects with increased susceptibility to emotional distraction (WMI subgroup) and overlapped with the left AMY region showing increased response in these subjects when compared to those unaffected by emotional distraction (Non-WMI subgroup). Given the evidence that AI is characterized by increased distractibility and reduced ability to focus attention (Stanford et al., 2009), and that AI has been linked to impaired executive performance (Enticott et al., 2006; Pietrzak et al., 2008; Kam et al., 2012), the present results suggest that AI may be a general executive factor that contributes to increased sensitivity to emotional distraction. This interpretation is also supported by a recent ERP study, which found an association between increased AI and inefficient functioning of the conflict detection system in a continuous performance task (Kam et al., 2012).

In sum, the present findings regarding individual differences in the susceptibility to emotional distraction point to factors that affect both the basic emotional sensitivity and general executive control. Also, these factors are linked to neural changes indexing increased sensitivity in both basic emotion processing regions (AMY), associated with bottom-up effects, and higher-level executive regions (dlPFC), associated with top-down influences.

Noteworthy, dysfunctional alterations in factors influencing emotional sensitivity and susceptibility to emotional distraction, along with changes in the associated neural correlates, could play an important role in affective disorders, such as anxiety and depression. These phenomena are linked to dysfunctional interactions between emotion and cognition, in general, which may also influence the relationship between immediate and long-term effects of emotion on memory (see Foland-Ross and Gotlib, 2012; Hayes et al., 2012; Morey and Brown, 2012 in the present research topic). Anxiety-related disorders, such as post-traumatic stress disorder (PTSD), involve pathology of both emotion and memory, which is associated with dysfunctional alterations of both bottom-up (MTL) and top-down (PFC) neural systems (Morey et al., 2009; Hayes et al., 2011). For instance, frequently reported memory-related symptoms of PTSD, such as intrusive recollections of traumatic memories (Kaspi et al., 1995; Harvey et al., 1998; McNally, 2006), have been linked to dysfunctions of the basic MTL-based mechanism (Hayes et al., 2011) identified in healthy participants as being responsible for the memory-enhancing effect of emotion (Dolcos et al., 2004b). Also, symptoms of *hypervigilance* along with an overall heightened sensitivity to both threatening and non-threatening stimuli observed in PTSD patients (Grillon and Morgan, 1999; Peri et al., 2000), have been linked to alterations of PFC function, which may explain increased non-specific distractibility to both trauma-related and unrelated stimuli in these patients (Morey et al., 2009). Given that these phenomena co-occur in clinical conditions, such as PTSD and depression, their concomitant investigation with tasks assessing both immediate and long-term effects (on WM and EM, respectively) provides a seemingly promising research venue. Such within-subjects investigations would contribute to the elucidation of the link between enhancing and impairing effects of emotion on cognition by complementing the studies separately investigating these effects in clinical conditions (see Dolcos, 2013 in the present research topic). Overall, the present findings from healthy participants, along with evidence from clinical patients, highlight the importance of these issues and warrant further concomitant investigations of interactions between enhancing and impairing effects of emotion, in both normal and clinical conditions.

## Conclusion

In summary, using a novel paradigm in which EM targets were initially encountered as WM distracters, the present study provided evidence for a link between the immediate and long-term impact of emotion. The present study also highlights the role of individual differences in the impact of emotional distraction. The study generated four main findings, as follows. Regarding the relationship between the immediate impact of emotional distraction on WM and the long-term impact on EM, the study yielded two findings: (1) emotional pictures that disrupted face WM but enhanced subsequent EM were associated with increased AMY and HC activity coupled with reduced dlPFC activity; (2) trials in which emotion enhanced EM without disrupting WM were associated with increased vlPFC activity. Regarding the role of individual differences in the impact of emotional distraction, the study yielded two additional findings: (3) participants who were more susceptible to the WMI effect of emotion showed greater AMY increases and PFC reductions; (4) AMY activity increased and dlPFC activity decreased with measures of attentional impulsivity. Collectively, these findings demonstrate that the immediate impact of emotional distraction on WM and the long-term impact of emotion on EM are mediated by overlapping and dissociable neural systems, involving both ventral/bottom-up and dorsal/top-down mechanisms, and that the brain regions mediating these effects are specifically sensitive to modulations by individual differences. Understanding the mechanisms mediating the impairing and enhancing effects of emotion on cognition, in general, and on memory, in particular, offers potential insights into understanding affective disorders, such as anxiety and depression, where their interaction is *dys*functional.

## Conflict of Interest Statement

The authors declare that the research was conducted in the absence of any commercial or financial relationships that could be construed as a potential conflict of interest.
